# Quantification of protein Z expression in lung adenocarcinoma tissues and cells

**DOI:** 10.1186/s40064-016-2610-x

**Published:** 2016-07-11

**Authors:** Hong Wang, Fang Huang, Xue-Yi Pan, Ze-Bin Guan, Wen-Bing Zeng, Ming-Jie Li, Rui-Hao Zhang

**Affiliations:** Department of Hematology, The First Affiliated Hospital of Guangdong Pharmaceutical University, Guangzhou, 510080 Guangdong People’s Republic of China; Department of Hematology, The Sixth Affiliated Hospital of Guangzhou Medical University, Qingyuan People’s Hospital, Qingyuan, 511518 Guangdong People’s Republic of China

**Keywords:** Lung adenocarcinoma, Protein Z, A549, 16-HBE, PZI

## Abstract

As a regulator of coagulation, abnormal Protein Z (PZ) expression may lead to the formation of blood clots in humans. While previous studies have shown that PZ protein is altered in several types of cancer, however, additional observations are needed to understand the complex biology involved. Herein, we investigated local alterations in PZ expression in lung adenocarcinomas by measuring gene and protein expression in both cancerous and normal lung tissues. Twenty-two (22) specimens of lung adenocarcinoma and 22 specimens of normal lung tissues from human patients were compared for the expression of PZ. In addition, A549 adenocarcinoma cells were compared to a normal epithelial cell line, 16-HBE, for in vitro PZ expression. In tissues and cells, PZ protein and gene expression were determined using western blot, immunohistochemistry and PCR. Lung adenocarcinoma tissues showed elevated expression of both PZ mRNA and protein compared with healthy tissue. Only protein expression was increased in cultured cell lines, which holds implications for the dominant source of PZ in tissues, as well as protein modifications necessary for PZ function. Protein Z appears to be associated with the presence of lung adenocarcinoma and may be a viable prognostic biomarker for lung cancer.

## Background

Protein Z (PZ) is a vitamin K-dependent (VKD) glycoprotein that acts as a potent cofactor for the enzyme protein Z-dependent protease inhibitor (ZPI). Together, these proteins mediate thrombin activation and blood coagulation by strongly inhibiting the activation of Factor Xa (FXa). PZ shares structural similarities with other VKD factors such as coagulation factor VII, IX, X, protein C and protein S, but holds no intrinsic enzymatic activity (Broze and Miletich 1984). While the specific mechanism of PZ’s actions in the anticoagulation cascade is not established, several reports demonstrate the formation of a PZ/ZPI complex that inactivates FX, thereby inhibiting the coagulation cascade prior to formation of the prothrombin complex. In fact, the presence of PZ enhances the inhibition of FXa by ZPI by 1000-fold (Fujimaki et al. 1998). In this regard, the traditional role of PZ in mediating anticoagulation is to serve as a critical factor in ZPI and FX signaling (Tabatabai et al. 2001), ultimately controlling thrombus formation.

Thromboembolisms are one of the most common ailments in cancer patients, as many hemostatic complications can arise due to malignancy (Wojtukiewicz et al. 2001). An imbalance in pro- and anti-coagulation factors may lead to blood coagulation abnormalities and vascular disorders, which could further exacerbate cancer progression, as well as attenuate immunologic defense mechanisms and therapeutic efficacy of chemotherapeutic drugs and other treatments (Sierko et al. 2012b; Wojtukiewicz et al. 2007). The activation of FX has been considered the most important step leading to the formation of thrombin and fibrin in both normal and pathologic conditions (Bick 1992; Falanga and Rickles 1999; Zacharski et al. 1992). However, it remains unclear whether disruption of inhibition of FXa by PZ/ZPI specifically underlies the pathogenesis of thrombosis and other blood coagulation disorders in cancer patients. Our previous studies have found that as malignant tumors progress, plasma levels of PZ significantly decrease (Shang et al. 2005), which indicates that PZ may be a factor contributing to poor prognosis in cancer patients. It remains unclear, however, whether circulating concentrations or local tissue PZ expression is primarily responsible for the downstream increase in thrombotic episodes in patients. Abnormal tissue expression of PZ has been observed in human cancers of the lung, breast, colon, and stomach (Sierko et al. 2011, 2012a, b, 2014). Interestingly, patients with these types of cancers are also more prone to developing thrombosis.

Determining mechanisms by which local changes in a tumor microenvironment lead to systemic changes in an individual is an important aim in cancer research. In the current study, we investigate PZ expression in lung adenocarcinomas, which is highly metastatic and likely to cause the development of thrombosis. We examined the expression of PZ in cell cultures and in lung adenocarcinoma biopsies using Western blotting, immunohistochemistry, and RT-PCR to observe any differences in PZ in the local tumor environment compared to normal tissue.

## Subjects and methods

### Specimen collection

This study protocol adhered to the principles of Helsinki and was confirmed by the ethics committee of the First Affiliated Hospital of Guangdong Pharmaceutical University. A total of 22 specimens were collected from pathologically-confirmed lung adenocarcinoma patients who underwent surgery in the Thoracic Department of the First Affiliated Hospital of Guangdong Pharmaceutical University between 2008 and 2011. Informed consent was obtained from all subjects before the study. The average age of the patients was 59, with 12 males and 10 females ranging from 27–80 years old. The cancerous tissue was collected from a primary lesion, avoiding the necrotic and inflammatory sites, while normal lung tissues were collected >5 cm, away from the corresponding lesion. Upon surgical removal, all specimens (0.5 cm × 0.5 cm × 0.2 cm) were immediately frozen in liquid nitrogen and stored at −80 °C until use.

### Cell culture

Human bronchial epithelial 16-HBE cells and a lung adenocarcinoma epithelial cell line, A549, were provided by the Experimental Medical Research Center at the Guangzhou Medical University. Cells were grown in DMEM supplemented with 10 % FBS at 37 °C in 5 % CO_2_ and passaged twice. Cultures with a density of 5 × 10^5^/ml were used for all experiments.

### Primer design

Primers were designed using Primer 6.0 software, synthesized by Sangon Biotech (Shanghai, China), and amplified a 159 bp product. Sequences are shown in Table 1.

**Table 1:** Real time PCR primer Sequence (5′–3′)

PZ	Forward: 5′-GCCCTCCATCGTGTGGAGCC-3′
	Reverse: 5′-TAAGCTTTCCTGGACGCCTGTGC-3′
GAPDH	Forward: 5′-AAGAGAGGCATCCTCACCCT-3′
	Reverse: 5′-TACATGGCTGGGGTGTTGAA-3′

### Tissue RNA extraction

Total RNA was extracted according to the manufacturer’s instructions using TRIzol reagent, and precipitated RNA was dissolved in 50 μl DEPC-treated water. Agarose gel electrophoresis showed three clear bands at 5, 18, and 28S. The absorbance of RNA samples at 260 and 280 nm was determined using UV spectroscopy and only samples with A260/A280 ratio between 1.8 and 2.0 were used for reverse transcription.

### qRT-PCR

cDNA was synthesized with 4 μl total RNA using the PrimeScript TM RT Master Mix Kit (Takara) according to the manufacturer’s instructions. Primer sets for PZ and GAPDH are listed in Table 1 (see “Primer design” section). By using a real time PCR machine (MX3000P, Stratagene, USA), PCR reactions were performed in a total of 20 μl reaction volume containing 10 μl 2 × Mix, 0.4 μl 50 × Rox, 3 μl (F + R) primer (2 μmol/L), 1 μl cDNA, and distilled water to make up to 20 μl. The thermocycling conditions were as follows: 30 s at 95 °C, followed by 40 cycles of 5 s at 95 °C, 30 s at 56 °C, and ended with 30 s at 72 °C. The quantification of gene expression changes were calculated as follows: (1) fold change = 2 − ΔΔCt; (2) ΔΔCt = ΔCt treatment group − ΔCt control group = (CT target gene − CT reference gene) treatment group − (CT target gene − CT reference gene) control group.

### Immunohistochemistry

Immunohistochemical analysis was performed using the Histostain-SP IHC Kit (Life Technologies) according to the manufacture’s protocol. Briefly, tissue samples were fixed in 10 % formalin, dehydrated, and paraffin-embedded. Paraffin blocks were cut for 4 μm-thick serial sections, and antigen retrieval was performed by microwaving in a pH 8.0 sodium citrate solution. Monoclonal mouse anti-human PZ antibody (Abcam, UK) (1:1600) was used as primary antibody, followed by incubation with ALP-conjugated goat anti-mouse IgG (1:5000), and DAB was used as the chromogen. PZ-positive cells were defined as cells with brown precipitates in the plasma. Cells without staining were marked as negative (−); cells stained light brown were marked as weakly positive (+); cells stained dark brown were marked as strongly positive (+++); and cells stained between weakly and strongly positive were marked as moderately positive (++). Staining was scored using a 13-point scoring system (Sierko et al. 2014) based on the percentage of stained cells (no cells stained = 0, <10 % = 1, 10–50 % = 2, 51–80 % = 3, >80 % = 4) and the intensity of the staining (negative staining = 0, weak staining = 1, moderate staining = 2, strong staining = 3). The final score was determined by multiplying the percentage of positively stained cell score with the intensity score (see Table 2 for a summary). All results were analyzed by two observers that were blinded to the condition of the patients.

**Table 2:** Immunohistochemical results of PZ in lung adenocarcinoma and healthy lung tissue

	Adenocarcinoma tissue	Healthy lung tissue
Proportion of positive cells (%)	>75	<25
Staining scores	58	9
Staining results	22/22 (100 %)	7/22 (31 %)

### Western blotting

Total protein was extracted from the frozen tissue samples using ultrasound homogenization with SDS lysis buffer (Beyotime Biotechnology, China). Lysates were cleared by centrifugation at 4 °C. The collected supernatants were measured for protein concentration using a BCA protein assay before being separated on 10 % SDS-PAGE gels and transferred to PVDF membranes. The membranes were incubated overnight at 4 °C with anti-PZ antibody (Abnova Taiwan, 1:2500) in PBS with 1 %BSA. The next day, membranes were washed and then incubated for 2 h at room temperature with ALP-conjugated secondary antibody (1:20,000). Protein signals were detected using NBT/BCIP (Beyotime Institute of Biotechnology) and analyzed with IPP software. The PZ/actin ratio was used for statistical analysis.

### Statistical analysis

All data are expressed as mean ± standard deviation. Statistical analysis was performed using SPSS 19.0 by T test, and p value <0.05 was considered statistically significant.

## Results

### mRNA expression of PZ gene in cells

**Fig. 1:**
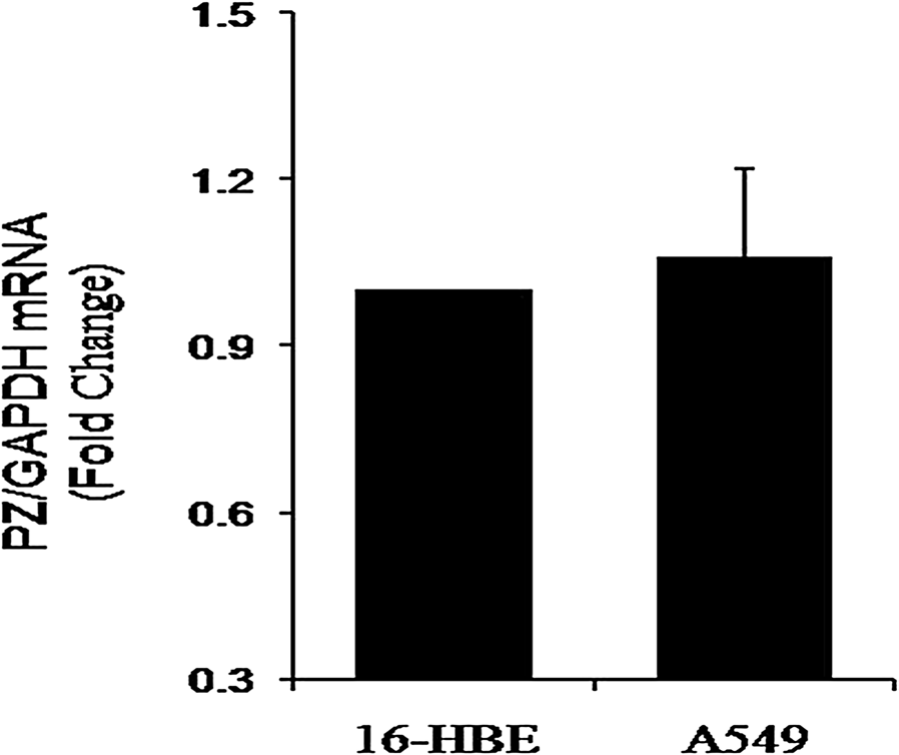
PZ mRNA in cell cultures. In comparison with normal cells, A549 adenocarcinoma cells exhibited a 1.06-fold increase in PZ mRNA expression. (P = 0.086)

We used SYBR-based quantitative fluorescence PCR to detect the expression of PZ gene in the A549 lung adenocarcinoma cell line and 16-HBE normal epithelial line, using GADPH to normalize the samples. The dissociation curves for both genes showed a single peak, suggesting that no off-target products, such as primer dimers, were formed. A549 adenocarcinoma cells exhibited a 1.06-fold increase in PZ mRNA expression over 16-HBE cells, but this increase was not statistically significant (Fig. 1).

### mRNA expression of PZ in human lung adenocarcinoma tissue samples

**Fig. 2:**
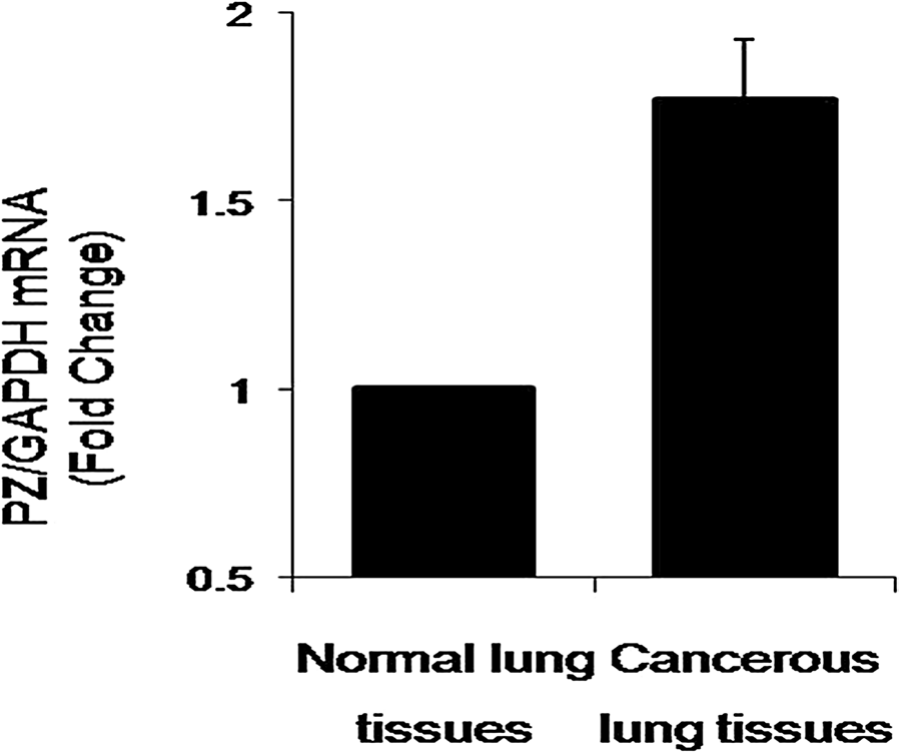
PZ mRNA in normal vs. cancerous tissue biopsies. Compared to normal tissue, PZ mRNA expression was 1.77-fold higher in lung adenocarcinoma tissue, which was statistically significant (P = 0.023). The altered PZ expression in adenocarcinoma patient samples, compared with normal tissues, suggests its involvement in the pathology of lung adenocarcinoma

Similarly, we used SYBR-based quantitative fluorescence PCR to detect the genetic expression of PZ in 22 lung adenocarcinoma and 22 normal tissues. A single peak was again observed, indicating that no off-target products such as primer dimers were formed. Compared with normal tissue, PZ mRNA expression was 1.77-fold higher in lung adenocarcinoma samples, which was statistically significant (Fig. 2).

### Immunohistochemical analysis of PZ in human lung adenocarcinoma tissue samples

**Fig. 3:**
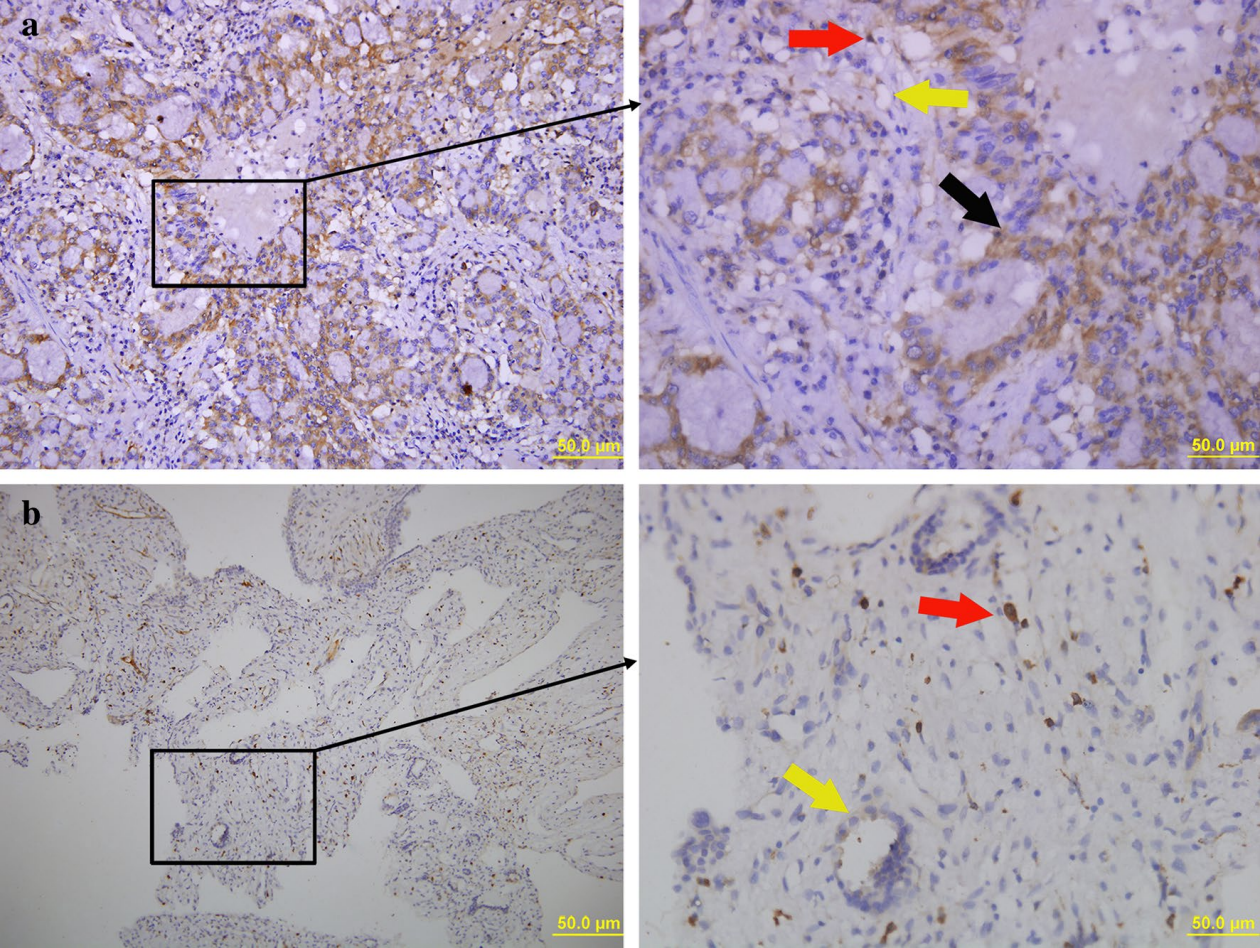
Representative images from immunohistochemical staining. In lung adenocarcinoma tissue (**a**), an increase in PZ staining (*brown*) is observed. The *black arrow* indicates the cytoplasmic expression of PZ, while the *red arrow* and *yellow arrow* indicates the presence of PZ protein in macrophages and endothelial cells, respectively. *Scale bar* 50 μm. **b** PZ protein was not expressed in the cytoplasm of cells in healthy lung tissue. The positively stained cells are macrophages (*red arrow*) and endothelial cells (*yellow arrow*). *Scale bar* 50 μm

The expression of PZ in cancerous lung tissue was increased, as indicated by intense brown staining in Fig. 3a. In contrast, healthy lung tissues showed minimal expression of PZ (Fig. 3c). Overall, PZ protein appears to be expressed in macrophages and endothelial cells in the newly formed vessels, which is in line with previous findings (Sierko et al. 2012b).

### Protein expression of PZ in A549 and 16-HBE cells

**Fig. 4:**
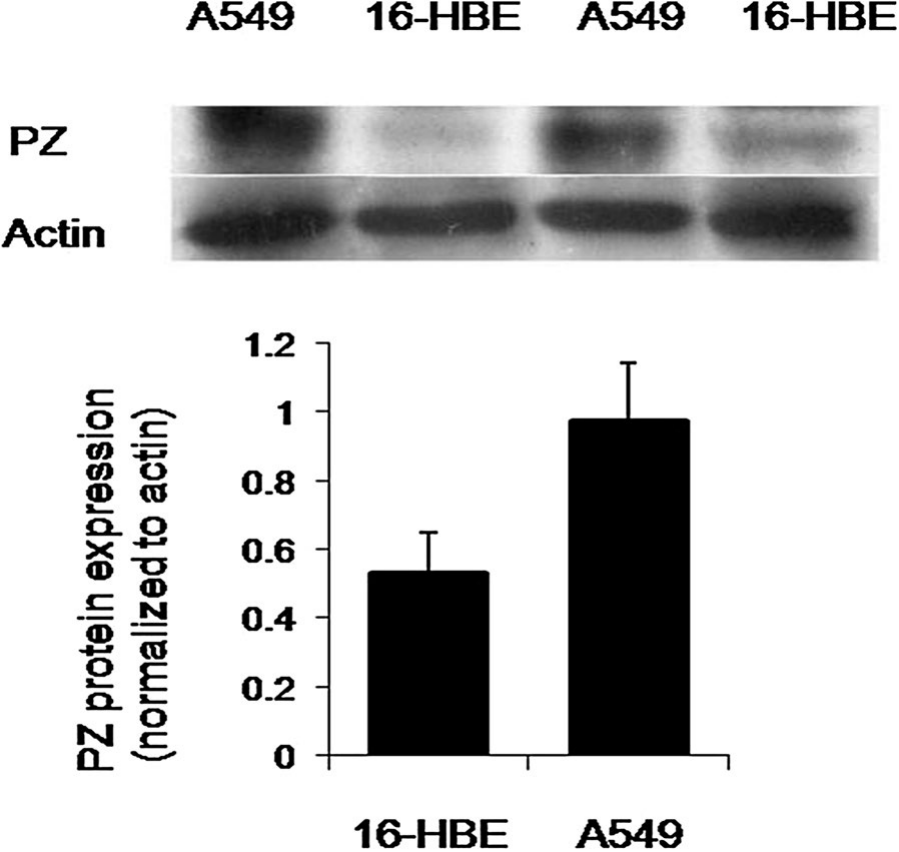
PZ protein expression is increased in adenocarcinoma cells. Representative images illustrate that Western blotting of isolated cells revealed a significant increase in PZ protein in A549 (adenocarcinoma) cells compared to normal cells (16-HBE). Samples were normalized to actin and quantitated (*P = 0.017)

As shown in Fig. 4, western blot analysis shows a significant 1.84-fold higher expression of PZ protein in A549 cells than 16-HBE epithelial cells. This suggests that the expression level of PZ protein may be associated with cancerous cells. Furthermore, this change is likely to be mediated by post-transcriptional or post-translational modifications, as total PZ mRNA expression was minimally altered (Fig. 4).

### Protein expression of PZ in lung adenocarcinomas and healthy lung tissues

**Fig. 5:**
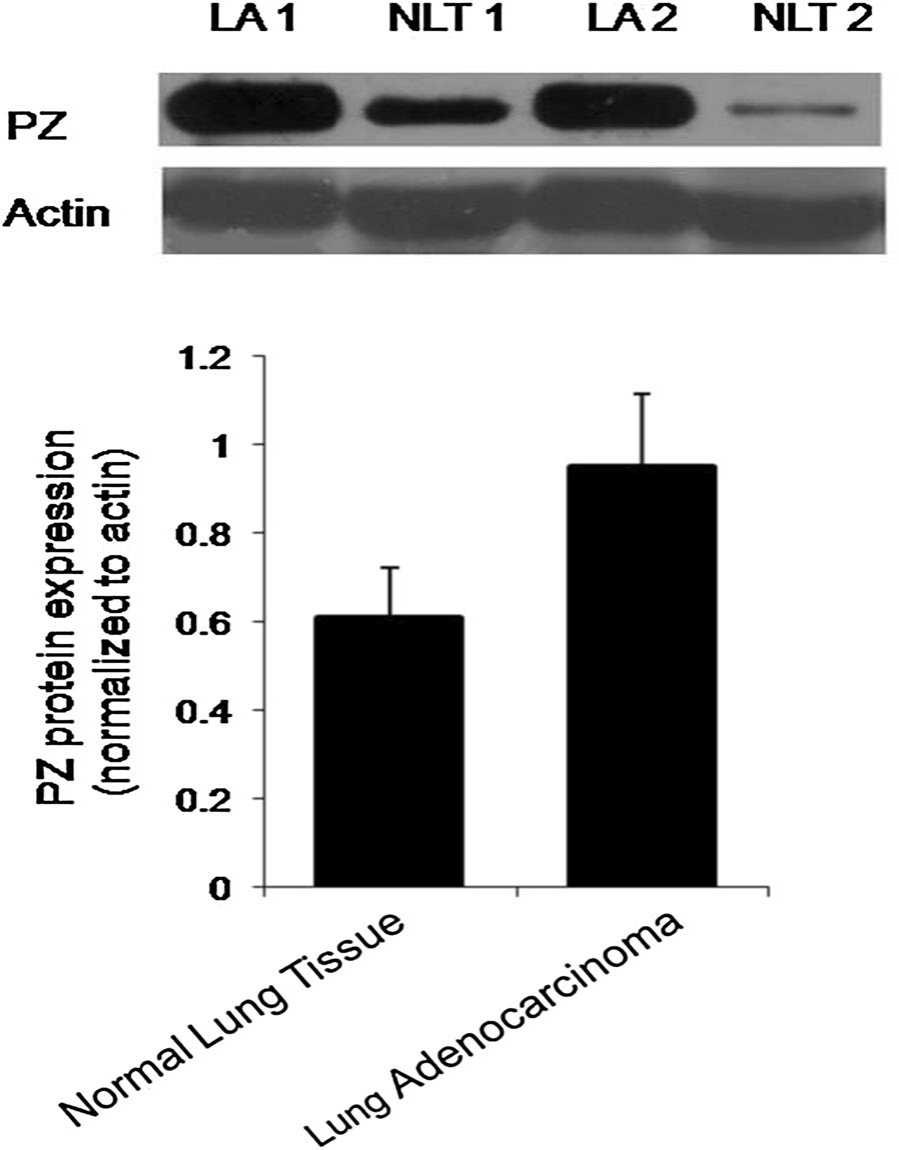
PZ protein expression is upregulated in cancerous tissue biopsies. Representative Western blots show that PZ expression is highly upregulated in lung adenocarcinoma (LA) tissue compared with normal lung tissue (NLT). Samples were normalized to actin and quantitated (*P = 0.014)

We next examined the expression of PZ protein in 22 lung adenocarcinoma and 22 healthy lung tissues via western blotting. Representative images of the membranes shown in Fig. 5 illustrate that PZ expression is significantly increased in cancerous tissues compared with normal lung tissue.

## Discussion

In order to examine whether protein Z (PZ) is altered in primary cancer tissues, we studied PZ expression in human lung adenocarcinomas, one of the most metastatic and thrombosis-prone lung cancers. Using Western blotting, immunohistochemical staining and real-time PCR techniques, we examined the expression levels of PZ in lung adenocarcinoma versus normal lung tissue. Our results show increased expression of PZ in lung adenocarcinoma tissues and higher expression in A549 than 16-HBE, which correlates with previous experimental results that provide a role for local PZ expression in the biology of several types of cancer (Sierko et al. 2011, 2012a, b, 2014). Although additional studies are needed, these results indicate that altered PZ expression plays a role in the local pathobiology of lung cancer, possibly distinct from its global role as an anti-coagulant.

Thromboembolisms are commonly associated with cancer malignancy, as global hemostatic changes are observed in response to tumor formation and growth. Thrombin and fibrin are largely known for their essential roles as coagulants in the blood. However, these and other related pro-coagulants, such as tissue factor and Factor X, have strong effects on cell biology aside from blood clot formation. Thrombin, for example, stimulates cancer cell proliferation and migration (Bick 1992; Zacharski et al. 1992), and acts on endothelial cells to stimulate angiogenesis and vascular permeability (Wojtukiewicz et al. 2001). Fibrin can act as a scaffold for balancing mechanical tension during tumor growth, or to bind growth factors that guide cell behavior or are released upon fibrin degradation (Zacharski et al. 1992).

Often, an increase in the expression of coagulation factors naturally leads to an increase in the expression of their inhibitors, which includes PZ (Sierko et al. 2012b). While evidence of direct effects of PZ on different cell types is minimal, studies have demonstrated a role for PZ in normal angiogenesis (Butschkau et al. 2014), accumulation of PZ around vascular lesions (Greten et al. 1998) and increased PZ expression surrounding blood vessels in breast cancer tissue (Sierko et al. 2011), all of which indicate a more direct involvement of PZ in endothelial and mural cell biology. Maintaining a balance between pro- and anti-coagulant factors is, therefore, not only critical for blood clot formation and degradation, but also for proper cellular activation and homeostasis within tissues themselves.

A relationship between PZ and cancer has been observed, but experimental results are conflicting and generally inconclusive. In liver cancer, it was found that PZ might serve as a tumor suppressor (Neumann et al. 2012). In acute leukemia, reduced plasma PZ levels were shown to increase the risk of bleeding in patients (Galar et al. 2012). Another recent study reported a significant drop of plasma PZ level in young acute lymphoblastic leukemia (ALL) patients undergoing induction therapy; however, this decrease did not correlate with bleeding or thrombosis (Cankal et al. 2013), indicating non-traditional roles for PZ in cancer cell biology. Sierko et al. recently showed an overexpression of PZ in breast, lung, colon, and stomach cancer tissues (Sierko et al. 2011, 2012a, b, 2014). Additionally, they detected elevated PZ expression in tissue-associated macrophages and endothelial cells in cancer microenvironments, and examined the expression and interactions of PZ, ZPI, prothrombin fragments (F1 + 2), and fibrin in non-small-cell lung carcinoma and colon cancer (Sierko et al. 2011, 2012a, b). Their findings suggest that PZ, through its anticoagulation properties, affects tumor angiogenesis, invasion and metastasis. On the other hand, our previous clinical studies have found that as a malignant tumor progresses, circulating levels of PZ significantly decrease (Broze and Miletich 1984). While this may seem conflicting to the data presented here and by others (Sierko et al. 2011, 2012a, b, 2014), it could simply mean that certain components of tumor (and tissue) microenvironments may not be properly represented in the circulation, and care should be taken during the establishment of truly representative novel circulating biomarkers.

## Conclusions

The current study confirms that PZ is locally present in lung adenocarcinomas. Given what is known about the biology of PZ, it may play a critical role in the tumor microenvironment, or in the systemic response to maintain homeostasis. We provide evidence that PZ should be studied to determine its distinct roles in tumor initiation, progression, angiogenesis and metastatic potential. It is possible that PZ may serve as an important target for treatments of lung adenocarcinomas and other tumors, and more experiments will help to determine the precise role of PZ in disease pathogenesis and progression.
